# The Effect of Hydroxamic Siderophores Structure on Acetylation of Histone H3 and Alpha Tubulin in *Pinus sylvestris* Root Cells

**DOI:** 10.3390/ijms20236099

**Published:** 2019-12-03

**Authors:** Joanna Mucha, Tomasz A. Pawłowski, Ewelina A. Klupczyńska, Marzenna Guzicka, Marcin Zadworny

**Affiliations:** Institute of Dendrology, Polish Academy of Sciences, Parkowa 5, 62-035 Kórnik, Poland; tapawlow@man.poznan.pl (T.A.P.); evelin@man.poznan.pl (E.A.K.); guzicka@man.poznan.pl (M.G.); zadworny@man.poznan.pl (M.Z.)

**Keywords:** histone, microtubule, pathogen, mycorrhizal fungi, Scots pine, iron chelating compounds

## Abstract

Protein acetylation affects gene expression, as well as other processes in cells, and it might be dependent on the availability of the metals. However, whether iron chelating compounds (siderophores) can have an effect on the acetylation process in plant roots is largely unknown. In the present study, western blotting and confocal microscopy was used to examine the degree of acetylation of histone H3 and alpha tubulin in *Pinus sylvestris* root cells in the presence of structurally different siderophores. The effect of metabolites that were produced by pathogenic and mycorrhizal fungi was also assessed. No effect was observed on histone acetylation. By contrast, the metabolites of the pathogenic fungus were able to decrease the level of microtubule acetylation, whereas treatment with iron-free ferrioxamine (DFO) was able to increase it. This latter was not observed when ferrioxamine-iron complexes were used. The pathogen metabolites induced important modifications of cytoskeleton organization. Siderophores also induced changes in the tubulin skeleton and these changes were iron-dependent. The effect of siderophores on the microtubule network was dependent on the presence of iron. More root cells with a depolymerized cytoskeleton were observed when the roots were exposed to iron-free siderophores and the metabolites of pathogenic fungi; whereas, the metabolites from mycorrhizal fungi and iron-enriched forms of siderophores slightly altered the cytoskeleton network of root cells. Collectively, these data indicated that the metabolites of pathogenic fungi mirror siderophore action, and iron limitation can lead to enhanced alternations in cell structure and physiology.

## 1. Introduction

Previous studies have demonstrated that iron plays a role in fungal-host interactions in both leaves [1,2,3] and roots [4,5,6,7,8]. Thus, metal-binding siderophores secreted by fungi, which regulate iron distribution, play an important role in virulence [1,3,5] and in maintaining the mutualistic interaction of mycorrhizae [9]. Studies on the bacterial hydroxamic siderophore showed the ability of pyoverdine to trigger induced systemic resistance [10,11,12]. The response of *Pinus sylvestris* roots to the application of different siderophores mirrored the response of the same roots to the application of the total metabolites secreted by either pathogenic or mycorrhizal fungi, and it was manifested as an imbalance in the distribution of elements that were measured in key cell compartments [13]. Iron and other mineral micronutrients are involved in many plant metabolic processes, including plant defense [14]. Different metabolic processes are induced in Scots pine in response to the entry of a fungus into root tissues, depending on the lifestyle/infection strategy of the invading fungus [15]. The type of metabolic processes that are induced in host tissues in response to an invading fungus has been suggested to be highly dependent on the acetylation/deacetylation of proteins in the plant host or fungus. In turn, many of the secreted fungal toxins, as well as resistance metabolites that are produced by the host, regulate the protein acetylation process. For example, trichostatin A, a derivative of hydroxamic acid, inhibits deacetylases, and thus provides a direct link between protein acetylation and microbial metabolites [16]. However, it is not known whether the acetylation in plants that is dependent on hydroxamic acid derivatives is also affected by siderophores produced by fungi. It is not known either whether siderophores depended acetylation processes result in plant death or regulate mutualistic symbiosis. It is of importance, as high levels of iron can promote cell death and contribute to the proliferation of necrotrophic pathogens [5], while also blocking the formation of a symbiotic relationship. This is particularly relevant, given that histone deacetylase activity, which is associated with the presence of iron, zinc, copper, magnesium, or calcium, has been documented in different phyla of organisms: animals, plants, and fungi [17]. As a result, the availability of cofactors likely affect changes in acetylation, among which iron is the strongest [18].

The dynamics of histone deacetylation that are associated with pathogen defense response [19,20,21,22] may not only promote the capacity of a pathogen to colonize a plant [23], but also suppress resistance in host tissues [19]. It appears that the level and the pattern of histone acetylation can be altered during the infection of host tissues by different pathogens [24]. However, the underlying mechanism of how this interaction is regulated remains an open question. A number of small secondary metabolites and non-ribosomal peptides produced by fungi, as well as several secondary metabolites that are synthesized by plants, have been suggested to act as deacetylation inhibitors [20]. The relationship between metabolites and histone deacetylase enzymes (mainly HDA6 and HAD19) and their mutual regulation have been corroborated, particularly in relation to fungal invasion and plant resistance [20]. Given the significance of the role of acetylation/deacetylation in plant/pathogen interactions, it is not surprising that several fungal toxins specifically target the deacetylation regulator proteins, histone deacetylases (HDACs). Additionally, a number of plant metabolites also target HDACs and, thus, play an important regulatory function in response to biotic stress. This might suggest that different histone deacetylase enzymes may exhibit a differential response to different fungi. Thus, the functional mechanism of these metabolites during fungal entry into a plant requires further investigation.

The complete inhibition of histone deacetylase, as along with strong inhibition of class I HDAC (HAD 6, HAD 7, HAD9, HDA17, HDA19), results in major changes in gene expression and can lead to cell death, and allows for plants to correct their response to biotrophic fungi, which require living plant tissue, and necrotrophic fungi, which kill host tissues and utilize the release of nutrients. One could hypothesize that the progression of necrotrophic pathogens would benefit from the inhibition of HDACs. Notably, different members of the histone deacetylase family may exert contrasting influences in cells in response to fungi and their metabolites [20,25,26].

Little information exists on the proteins, other than histones, which are regulated by different histone deacetylases in plants; although research in Arabidopsis has demonstrated that protein acetylation might occur in a variety of subcellular compartments [27,28,29]. Histone deacetylases (e.g., RPD3/HAD1-like, HD2 or SIR2-like) have been localized in the nucleus, but they can also be found in mitochondria, chloroplasts, endoplasmic reticulum, and cytoplasm [30]. The function of histone deacetylases (HDA14) located in the cytoplasm is, in turn, the acetylation of α-tubulin [28], which affects the conformation of microtubules. Microtubules also play an important role in triggering the defensive response in plants. Their re-orientation or depolymerisation has been observed during the initial stages of incompatible interactions [31]. Microtubules are engaged in intracellular communication in the course of pathogen-host interactions. Given these collective reports and the study by Dellagi et al. [32], a question can be raised regarding whether iron can also affect or regulate changes in the arrangement of microtubules that occur in host cells during host defense [33]. If a substantial portion of the microtubules in a cell us altered during a plant-fungal interaction, this might also have an impact on cell division.

Insight into host response affected by siderophores may thus help to identify the factors that determine the mutualistic or antagonistic nature of the plant-fungal interactions. Additionally, further research could also help to determine whether acetylation is involved in processes of fungal entry into host tissues. The objectives of the present study were to: (i) identify the degree of acetylation of histones and microtubules that occur during siderophore-cell interactions in plants. It is assumed that siderophores will affect the activity of deacetylases, since studies in animal cells indicate that iron affects deacetylases, which results in the decreased acetylation of histones and microtubules; (ii) identify the level of cell division. It is hypothesized that an increase in cell division will occur when the root tissues are treated with siderophores that have not been chelated with iron; and, (iii) assess the level of cell death, as it is plausible that siderophores will increase cell death. These questions were addressed by assessing the impact of three different families of iron-chelated and non-chelated hydroxamate siderophores on *P. sylvestris* root cells. The impact of crude preparations of metabolites of pathogenic and mycorrhizal fungi on *P. sylvestris* root cells was also examined.

## 2. Results

### 2.1. Assessment of Microtubule and Histone Acetylation

**Figure 1:**
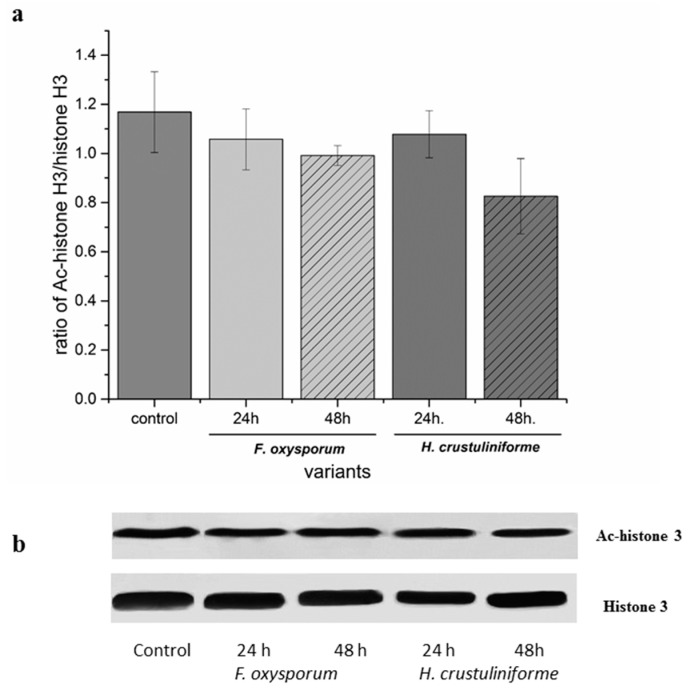
The effect of metabolites of pathogenic (*Fusarium oxysporum*) vs. mycorrhizal (*Hebeloma crustulinofrome*) fungi on histone acetylation in *P. sylvestris* root cells. Control roots were only treated with filter paper. (**a**) The proportion of acetylated histone H3 to non-acetylated histone H3. Bars and whiskers represent the mean ± SE, respectively. (**b**) Western-blot analysis of the effect of different siderophores on the proportion of acetylated histone H3 vs. non-acetylated histone H3 (the uncut gels can be found in the Supplementary).

**Figure 2:**
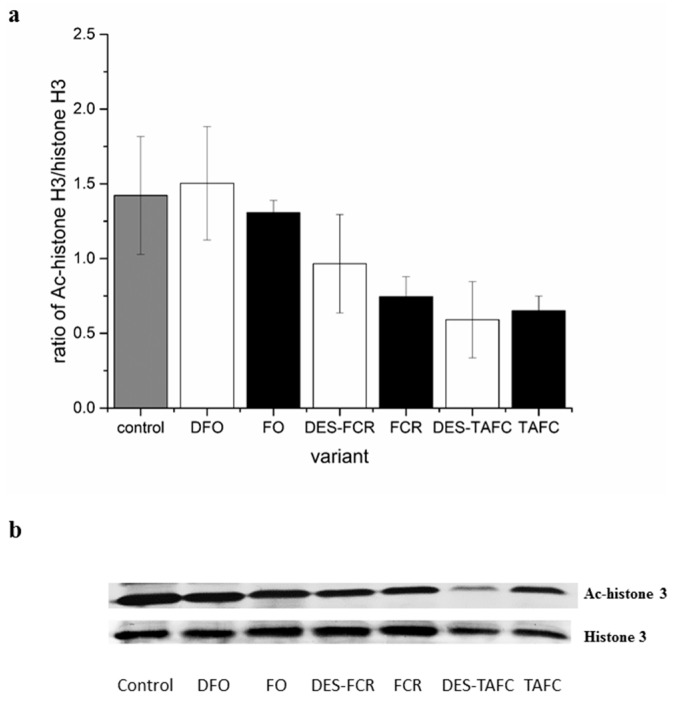
The effect of structurally different siderophores (ferrioxamine—FO, ferricrocin—FCR and triacetylfusarinine C—TAFC) chelated with iron and their iron free forms (desferrioxamine—DFO, desferricrocin—DES-FCR and destriacetylfusarinine C—DES-TAFC) on histone acetylation in *P. sylvestris* root cells. Control roots were treated with distilled water. (**a**) The proportion of acetylated histone H3 to non-acetylated histone H3. Bars and whiskers represent the mean ± SE, respectively. (**b**) Western-blot analysis of the effect of different siderophores on acetylated histone H3 and non-acetylated histone H3 (the uncut gels can be found in the Supplementary).

Metabolites that were secreted by the pathogenic fungus, *Fusarium oxysporum*, and the mycorrhizal fungus, *Hebeloma crustuliniforme* did not appear to have statistically significant effect on the level of histone acetylation, based on histone H3 acetylation (Figure 1), as determined by the analysis of western-blots (immunoblots). The results indicated that the siderophore treatments had the greatest effect on histone acetylation (Table 1). A more pronounced degree of histone deacetylation was observed in roots that were treated with triacetylfusarinine as opposed to the level of histone acetylation that was observed after treatment with ferrioxamine. No statistically significant effect was observed based on iron binding capacity and its interaction with a siderophore (Figure 2, Table 1).

**Figure 3:**
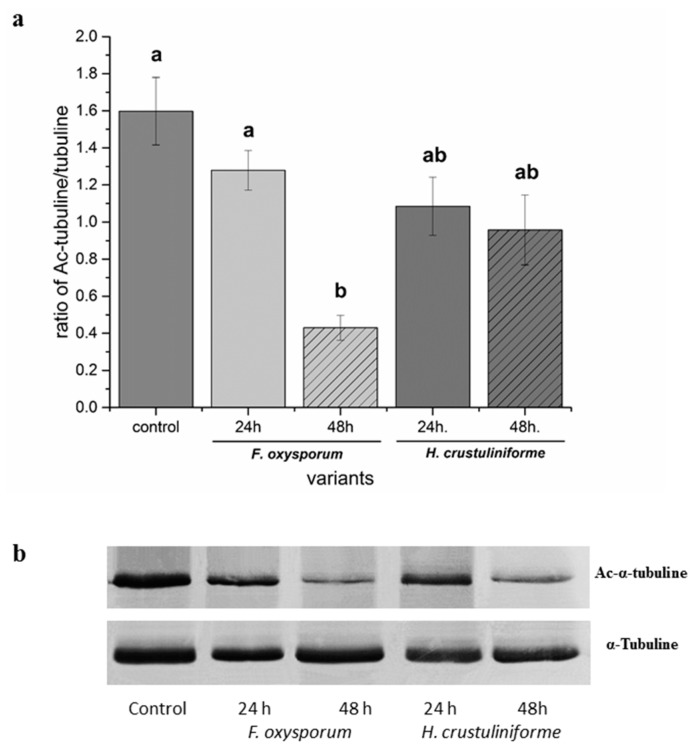
The effect of metabolites of pathogenic (*Fusarium oxysporum*) vs. mycorrhizal (*Hebeloma crustuliniforme*) fungi on tubulin acetylation in *P. sylvestris* root cells. Control roots were only treated with filter paper. (**a**) The proportion of acetylated α-tubulin to non-acetylated α-tubulin. Bars and whiskers represent the mean ± SE, respectively. Values with different letters are significantly different (*p* < 0.05) according to the Tukey test. (**b**) Western-blot analysis of the effect of different siderophores on acetylated α-tubulin and non-acetylated α-tubulin (the uncut gels can be found in the Supplementary).

**Figure 4:**
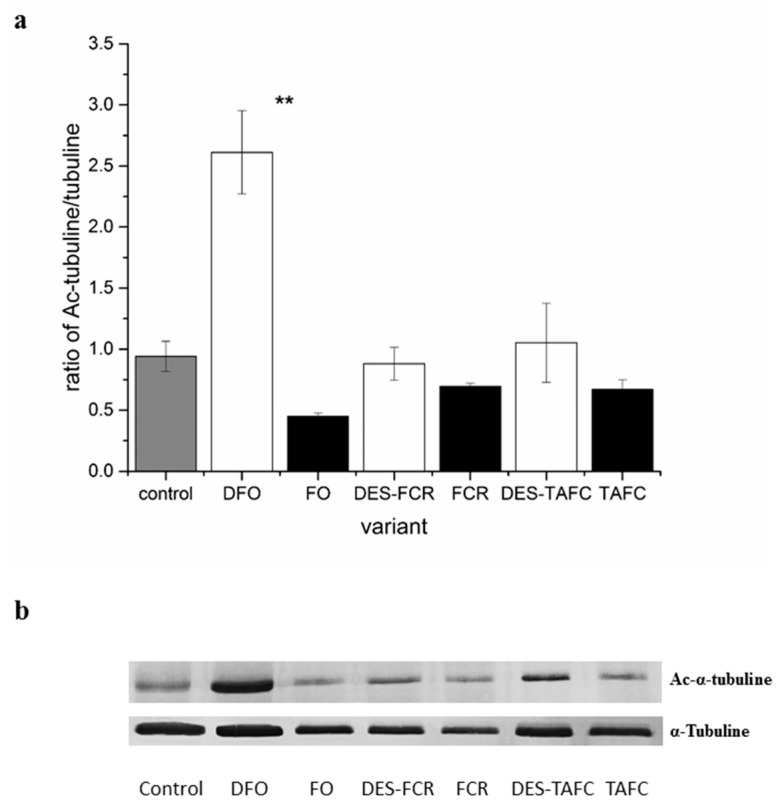
The effect of structurally different siderophores (ferrioxamine—FO, ferricrocin—FCR and triacetylfusarinine C—TAFC) with chelated iron, and their iron free form (desferrioxamine—DFO, desferricrocin—DES-FCR and destriacetylfusarinine C—DES-TAFC) on tubulin acetylation in *P. sylvestris* root cells. Control roots were treated with distilled water. (**a**) The proportion of acetylated α-tubulin to non-acetylated α-tubulin. Bars and whiskers represent the mean ± SE, respectively. Values with ** indicated statistically significant different means (*p* < 0.01) according to a Student *t*-test. (**b**) Western-blot analysis of the effect of different siderophores on acetylated α-tubulin and non-acetylated α-tubulin (the uncut gels can be found in the Supplementary).

Differences in tubulin acetylation were observed in response to the metabolites of pathogenic fungus although no differences in histone acetylation were observed in roots exposed to the metabolites of the pathogenic (*F. oxysporum*) or mycorrhizal fungi (*H. crustuliniforme*) (Figure 3). The highest level of acetylated α-tubulin was observed at 24 h after exposure of roots to the metabolites of the fungal pathogen, and the lowest level after 48 h. In contrast, no change in the ratio of acetylated to non-acetylated tubulin was observed when the roots were exposed to metabolites of the mycorrhizal fungus. Analysis of variance indicated a significant effect of two independent variables (type of siderophore and the ability of a siderophore to bind iron) on the proportion of acetylated to non-acetylated tubulin (Table 1). In general, a higher level of acetylated tubulin was observed in roots that were treated with desferrioxamine (DFO) than was observed with the siderophores, ferricrocin (FCR), or triacetylfusarinine C (TAFC). The ability to bind iron also contributed to increases in acetylated tubulin. A significant interaction of siderophore × ability to bind iron was also observed. In particular, the ability to bind iron had an impact on the level of tubulin acetylation that resulted from the treatment of roots with ferrioxamine (FO) (Figure 4).

### 2.2. Analysis of the Tubulin Cytoskeleton

**Figure 5:**
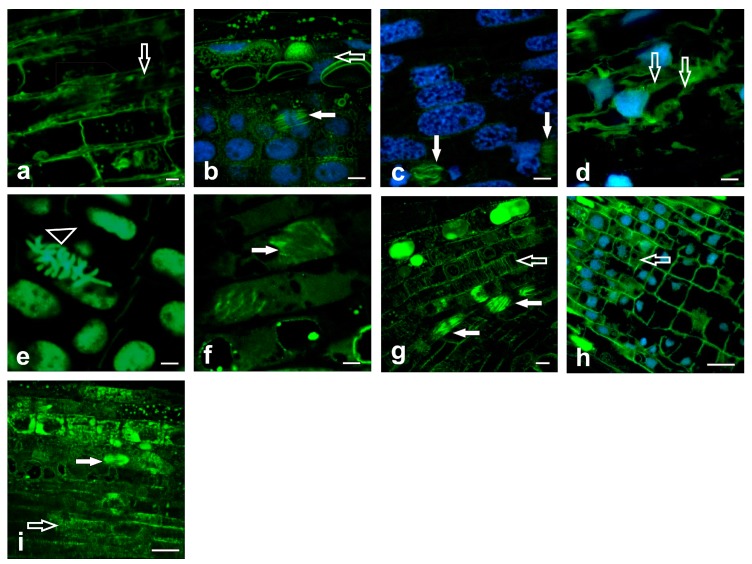
Confocal photomicrographs of the tubulin cytoskeleton in *P. sylvestris* root cells. Depolymerization of microtubules was observed in response to treatment of roots with metabolites of the pathogenic fungus, *F. oxysporum* (bar = 10 µm) (**a**); Rare depolymerization of the microtubule network in cells of the outer part of the parenchymatic cortex in response to treatment with metabolites of the mycorrhizal fungus, *H. crustuliniforme*. Note the visible array of undisturbed mitotic spindles (bar = 10 µm) (**b**); disorder of mitotic spindle morphology after application of ferrioxamine—FO (bar = 7.5 µm) (**c**); a complete disruption of the microtubules and disintegration of the cytoskeletal network after application of desferrioxamine—DFO (bar = 7.5 µm) (**d**); complete disassembly of cortical microtubule arrays in response to ferricrocin—FCR (bar = 7.5 µm) (**e**) and aberrant pattern to its iron free form DES-FCR (bar = 7.5 µm) (**f**); well organized framework of microtubules exhibiting a transverse co-alignment and also spindle microtubules with an organization characteristic of different mitotic figures in response to DES-TAFC treatment (bar = 10 µm) (**g**); complete disruption of microtubules and their orientation into clusters of cytoplasmic fibers after application of TAFC (bar = 25 µm) (**h**); and, properly orientated kinetochore microtubules during spindle assembly are visible in control root cells of *P. sylvestris* (bar = 25 µm) (**i**). On average 90 optical sections get projected on one plane, and the section thickness tissue section reached c.a. 0.13 µm. Note that: arrows filled indicate microtubule of mitotic spindles, empty arrows indicate cortical microtubule, and triangle indicates chromosomes.

The appearance of the tubulin cytoskeleton was assessed while using confocal microscopy. The exposure of roots to metabolites of the pathogenic fungus, *F. oxysporum*, resulted in a complete depolymerization of the microtubule network (Figure 5a). In contrast, exposure to metabolites secreted by the ectomycorrhizal fungus, *H. crustuliniforme*, resulted in the diffuse depolymerization of microtubules in only a few cells in the outer portion of the root cortex containing parenchymatic cells. The cytoskeleton network appeared to be normal in most cells, including both cortical and spindle microtubules, in which a visible array of normal mitotic spindles was observed (Figure 5b). The appearance of the microtubule network was related to the availability of iron. The exposure of roots to FO resulted in the appearance of disordered microtubule morphology in root cortical cells and in the mitotic spindles, but not complete disappearance (Figure 5c). In contrast, plants that were exposed to the iron-free form of FO exhibited a complete disruption of microtubules and disintegration of the cytoskeleton network (Figure 5d). The complete rearrangement of cortical microtubule arrays was observed in response to both FCR and its iron free form (Figure 5e,f). A total disassembly of the microtubule cytoskeleton was observed in response to the FCR treatment (Figure 5e), although in select cells of roots in response to treatment with the iron-free form of FCR the aberrancy of microtubule arrays was also observed (Figure 5f). In general, these observations contrasted with the observations of roots cells of *P. sylvestris* that were treated with destriacetylfusarinine C (DES-TAFC), where a well-organized framework of microtubules exhibiting transverse coalignment, as well as spindle microtubules with organization characteristic of different mitotic figures (Figure 5g), were readily observed. In contrast, the application of TAFC induced an intense reorganization of the cortical microtubules, being evidenced as a complete disruption of the microtubules and microtubule orientation into clusters of cytoplasmic fibers (Figure 5h). Different array orientations of the cortical microtubules were observed in the control cells of *P. sylvestris* roots that were reflective of the cell’s growth axis (Figure 5i). Properly orientated microtubules during spindle assembly were also visible in the control cells (Figure 5i).

**Figure 6:**
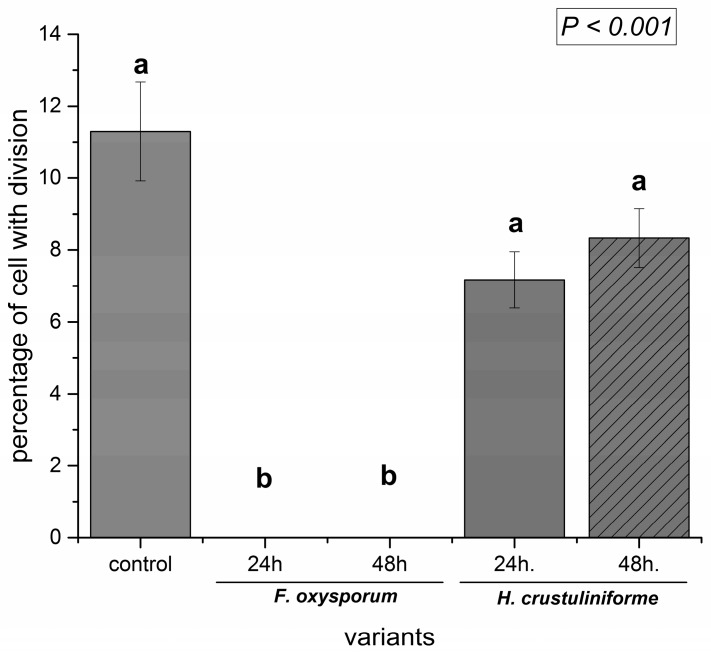
Effect of fungal metabolites of pathogenic (*Fusarium oxysporum*) vs. mycorrhizal (*Hebeloma crustuliniforme*) fungi on the level of cell division in *P. sylvestris* root cells. Control roots were only treated with filter paper. Bars and whiskers represent the mean ± SE, respectively. Values with different letters indicate statistically significant different means (*p* < 0.05) according to the Tukey test.

**Figure 7:**
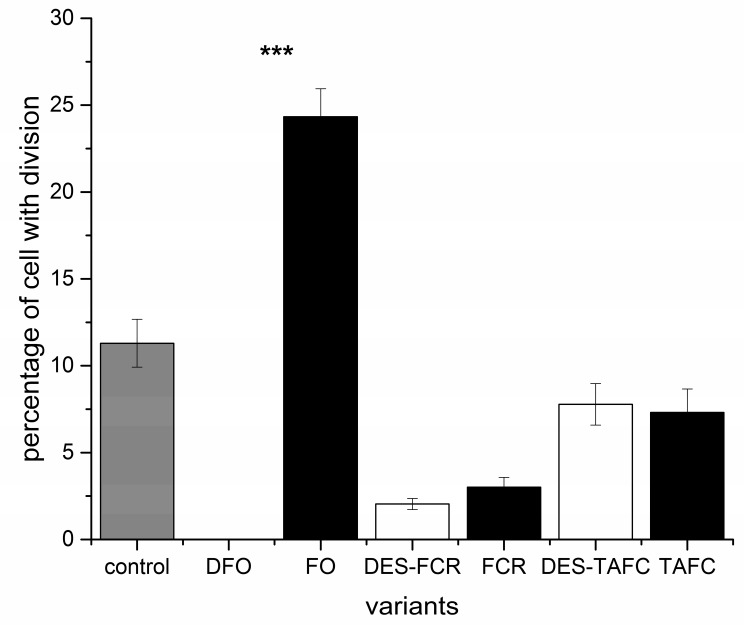
The effects of structurally different siderophores (ferrioxamine—FO, ferricrocin—FCR and triacetylfusarinine C—TAFC) with chelated iron and their iron free forms (desferrioxamine—DFO, desferricrocin—DES-FCR and destriacetylfusarinine C—DES-TAFC) on the level of cell division in *P. sylvestris* root cells. Bars and whiskers represent the mean ± SE, respectively. Values with *** indicate statistically significant different means (*p* < 0.001), according to a Student *t*-test.

The proportion of dividing cells that was present in root tissues was also determined. The results indicated that the metabolites that were produced by the pathogenic fungus inhibited cell division in *P. sylvestris* root cells and a level of cell division exposed to metabolites of the mycorrhizal fungus (about 8%) was similar to the control tissues (about 11%) (Figure 6). A two-way analysis of variance indicated that the type of siderophore significantly affected cell division (*p* < 0.001). The greatest inhibition of cell division was observed in roots that were treated with FCR and iron-free form of ferrioxamine (DFO), and the least inhibition with FO (Figure 7). The ability to bind metals also contributed to the observed effect on cell division (*p* < 0.001), although this effect was dependent on the type of siderophore. For example, a significant siderophore × metal binding ability interaction (*p* < 0.001) occurred. Differences in the level of inhibition of cell division between iron-chelated and iron-free forms of a siderophore were observed for ferrioxamine (Figure 7).

### 2.3. Cell Death Assessment (Nuclear DNA Fragmentation)

**Figure 8:**
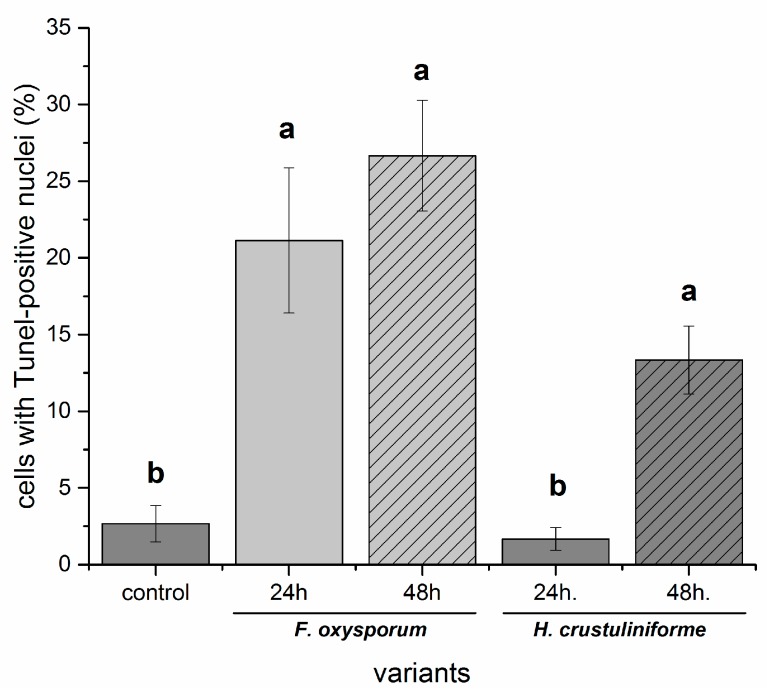
The effect of fungal metabolites of pathogenic (*Fusarium oxysporum*) vs. mycorrhizal (*Hebeloma crustuliniforme*) fungi on the number of cell tunel positive-nuclei in *P. sylvestris* root cells. Control roots were only treated with filter paper.

**Figure 9:**
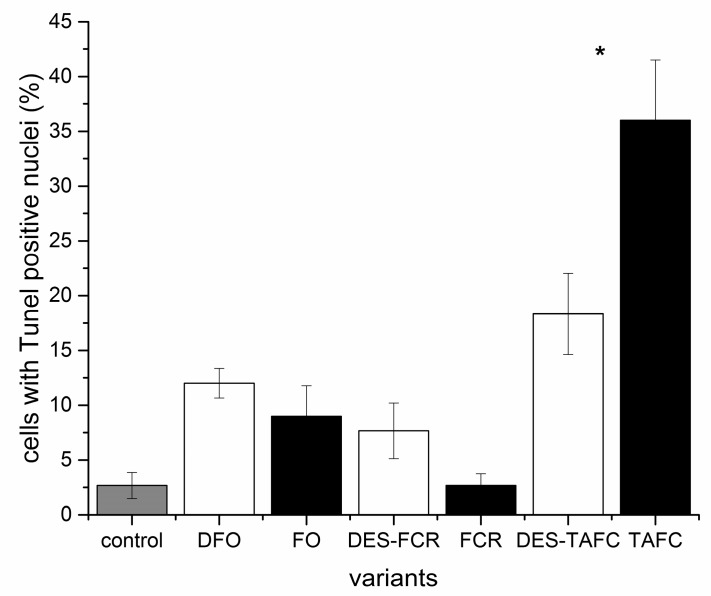
The effect of structurally different siderophores (ferrioxamine—FO, ferricrocin—FCR and triacetylfusarinine C—TAFC) with chelated iron and their iron free forms (desferrioxamine—DFO, desferricrocin—DES-FCR and destriacetylfusarinine C—DES-TAFC) on the number of cells with a tunel-positive nucleus in *P. sylvestris* root cells. Bars and whiskers represent mean ± SE, respectively. Values with * indicate significantly different means between DES-TAFC and TAFC (*p* < 0.05), according to the Student *t*-test.

**Figure 10:**
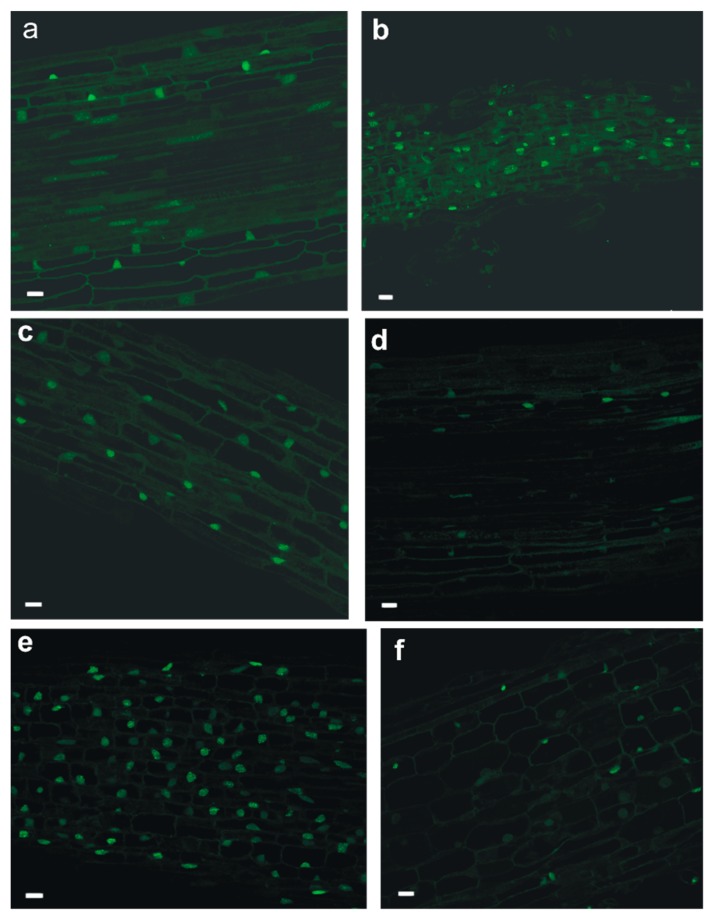
Tunel staining of cells of *P. sylvestris* root in respons to treatment of: desferrioxamine (DFO) (**a**); ferrioxamine (FO) (**b**); desferricrocin (DES-FCR) (**c**); ferricrocin (FCR) (**d**); destriacetylfusarinine C (DES-TAFC) (**e**); and, triacetylfusarinine C (TAFC) (**f**). Bar = 25 µm.

The proportion of cells with Tunel-positive cell nuclei was greater in roots that were exposed to the metabolites of the pathogenic fungus than mycorrhizal fungus and it increased over time in *P. sylvestris* roots that were exposed to the metabolites of the latter one (Figure 8). The greatest impact was associated with the treatment of roots with TAFC relative to the other two other siderophores tested though the ability to bind iron was the variable that affected the number of Tunel-positive cells in *P. sylvestris* roots treated with TAFC (Figure 9 and Figure 10).

## 3. Discussion

Despite increasing interest in the involvement of iron in the early stages of plant infection by a fungal pathogen, knowledge regarding this subject is insufficient, and the majority of studies have not focused on gymnosperms, a globally dominant group of many of the harshest terrestrial environments occupied by woody plants [2,5,32]. Information regarding the role of siderophore involvement in the regulation of entry into host root tissues by pathogenic vs. mycorrhizal fungi is especially lacking. Previous studies have established that siderophores induce an iron imbalance in different organelles of root cells of *Pinus sylvestris* [13], although bacterial hydroxamic siderophore (pyoverdine) act through plant iron disbalance [34]. In the current study, we assessed whether the iron binding activity of siderophores impacted the acetylation of histones and microtubules.

Previous studies reported diverse roles for the acetylation/deacetylation of histones during plant-pathogen interactions [19,23]. The activity of histone deacetylases that decrease histone acetylation requires the binding of metals, such as iron [18]. However, in our present study, the lack of changes in histone acetylation was observed in response to metabolites of pathogenic or mycorrhizal fungi relative to the levels of acetylation that were observed in control tissues (Figure 1). Although iron is an important cofactor in histone deacetylase activity, changes in the levels of other metal ions may also correlate with its activity [17,18]. Blocking the activity of metalloenzymes by other than iron metals, competing for the same binding site, such as copper, magnesium, and zinc [17], highlights the potential functional differences in elicitor components, and it suggests that the categorization of siderophores into different functional groups might be crucial in understanding the process of fungal invasion. Indeed, correlated changes between iron and other metals were previously observed in the nuclei of cells that were exposed to TAFC [13]. Studies on bacterial hydroxamic siderophore support the conclusion that difference in ability to induce iron imbalance by various pyoverdines is the cause of differences in triggering resistance mechanism mediated by jasmonic acid [10]. Thus, the simultaneous higher iron concentration [13] and decrease in acetylation, marks iron availability as essential factors determining the success of fungal invasion. The oxidation state in the case of iron can also inhibit deacetylases and other metalloenzymes, since only divalent iron or other metals are able to activate deacetylases [18].

Siderophores and the metabolites of both mycorrhizal and pathogenic fungi impact most of the elements in the cell wall and cytoplasm [13], and histone deacetylases, which are located in the cytoplasm, regulate the acetylation of microtubules and affect their conformation. Microtubules play an important role in triggering the defense response in plants, since their re-orientation and/or depolymerisation is observed during the initial stages of interactions of host resistant to specific pathogen [31]. Therefore, it was not surprising that, in the current study, depolymerization of microtubules was mainly observed in response to metabolites of the pathogenic fungus and only infrequently in response to the metabolites of the mycorrhizal fungus. Previous studies have reported that the reorganization of the cytoskeleton is characteristic of the entry of fungal pathogens with low specificity to *P. sylvestris* roots [33]. Thus, the host cytoskeleton represents a potential target for fungal virulence factors during pathogenic, as well as mutualistic, symbioses. In our study, a decrease in α-tubulin acetylation, depolimeryzation of microtubule network, as well as a decrease in cell division, was observed in *P. sylvestris* root cells in response to metabolites of the pathogenic fungus, *F. oxysporum*. The degradation of plant microtubule networks that inhibit protein secretion and ultimately suppress the cell-wall mediated defense response might be the consequence of tubulin acetylation that is caused by compounds extracellularly secreted by a pathogen [35], and the deacetylation of microtubules is connected with their depolymerization [36,37]. In contrast to the response to pathogens, mycorrhizal fungi require living and well-formed plant roots to establish a functional association. Microtubule acetylation is correlated with the presence of iron. Given that iron imbalance caused by hydroxamic siderophore pyoverdine might impact the trade-off between plant growth and immunity [34] in response to pathogen encountering, a plant can prioritize one process over the other [38]. We speculate delaying plant recognition of invading hyphae by secreting siderophores that bind plant iron is a principal mechanism of pathogens. Thus, we observed changes in acetylation and microtubule response (response in cytoplasm to environmental signals) responsible for rapid defense responses without involving nuclear mechanisms. Changes in the acetylation of tubulin by specific deacetylases actually regulates microtubule-dependent cell movement [39]. Cell polarization that is brought about by the microtubule network represents one of the first defense responses of plant cells to a pathogen, which cell death often follows [40].

Slightly different relationships between microtubule acetylation and organization, cell division, and cell death were observed in response to the used siderophores. While the level of acetylation was similar to the control, differences in the structure of the microtubule cytoskeleton and a number of observed cell divisions were related to the type of siderophore or whether it was non- or iron-chelated. This apparent paradox might be explained if an increase in acetylated tubulin levels can cause both microtubule destabilization and increase microtubule stability [36,37]. The results reported by Cheng et al. [41] indicated that increasing acetylated-tubulin levels did not affect patterns of cell division. In contrast to angiosperms, conifer species in the genus *Pinus* contain a high level of acetylated microtubules, especially within division structures during the mitosis of cells [36]. Acetylation of tubulin might actually take place in the cytoplasmic pool, and on microtubules, as observed by others [36,42]. Microtubule structure is also highly sensitive to calcium levels in the cytoplasm. Thus, siderophore might influence the stability of microtubules, not only by influencing acetylation, but also by directly impacting the level of available free calcium level. Generally, cytoskeletal network reorganization is achieved by a balance in the level of stable/dynamic microtubules that is closely dependent on a variety of post-translational modifications and associated protein microenvironments [43]. Although it can be assumed that fungal effectors and siderophores can affect the stability of the microtubule cytoskeleton, the action of siderophore might rather be related with binding various elements than with microtubule acetylation. Such conclusions also support research based on pyoveridine from *Pseudomonas putida* and *Pseudomonas fluorescens* that indicated that siderophore interfere in defence-related early singaling events but is poorly correlated with induced by this compound jasmonic acid based systemic resistance [12]. Therefore, the correlation between acetylation and microtubule stability and microtubule function needs to be further explored.

## 4. Materials and Methods

### 4.1. Organisms and Growth Conditions

The seeds of *Pinus sylvestris* that were used in the study were obtained from Bolewice, Western Poland (52°28′N and 16°03′E). The seeds were surface-sterilized with 0.1% HgCl_2_ (*w*/*v*; Polish Chemical Reagents, Gliwice, Poland), rinsed several times in sterile distilled water, and then germinated on 0.6% water agar (*w*/*v*; Difco, Fisher Scientific, Hampton, NH, USA). An agar-based growth medium [44] was poured into 14-cm (diameter) petri dishes. After solidification, half of the medium was removed and the remaining half was covered with filter paper (Whatman no. 1, Springfield Mill, UK) to prevent the roots from growing into the medium, but ensuring their access to nutrients. Five germinated seeds were placed on the filter paper in each petri dish and then covered with cellophane foil to prevent desiccation. After two weeks of growth under fluorescent lighting (Osram L36/W77 Flora; 100 µEm^−2^ s^−1^ for 16 h a day) at 60% relative humidity and a temperature regime of 24 °C during the day and 20 °C at night, the roots were treated with 0.5 mM solutions of the following siderophores for 24 h: desferrioxamine (the compound most often used in experiments involving iron limitation)—DFO, desferricrocin—DES-FCR and destriacetylfusarinine C—DES-TAFC. Iron-chelated siderophores were also applied as one of the experimental variants to assess the effect of iron limitation alone on plant roots (ferrioxamine—FO, ferricrocin—FCR and triacetylfusarinine C—TAFC). Iron free and iron-chelated forms of siderophores were purchased from EMC microcollections GmbH (Tübingen, Germany). The seedling roots treated with water that served as a control. Filter paper overgrown with a two-week old mycelial mat (necrotrophic pathogen *Fusarium oxysporum* or ectomycorrhizal fungus *Hebeloma crutuliniforme*) was placed on the roots of *P. sylvestris* seedlings under cellophane foil growing in the conditions previously stated to compare the response of *P. sylvestris* root cell to the different siderophore treatments with and on metabolites produced by fungi. Plant roots and fungal inoculants were physically separated by a Particle Track-etched Membrane (PTM; 10 µm thick, 0.2 µm size of the mesh; The Institute for Nuclear Chemistry and Technology, Warsaw, Poland) to allow for the migration of metabolites to the roots, but prevent hyphae from growing into the host roots. Preliminary studies have demonstrated that the cap layer of roots reaches a length of 0.5 cm from the root tip; thus, the first fragment cut from the root was considered as meristematic tissue. The experiments were repeated three times. For experiment arrangement please see Table S1.

### 4.2. Assessment of the Acetylation of Microtubules and Histones

The proteins were extracted from roots of pine seedlings that were exposed to the aforementioned treatments, as described by Staszak and Pawłowski [45] and Staszak et. al. [46,47]. After extraction, the proteins were subjected to electrophoresis (SDS-PAGE) while using 250 µL modified Laemmli Sample Buffer [48] at pH 6.8, containing: 62.5 mM Tris-HCl, 2% (*w*/*v*) SDS, 15 mg/mL of DDT and 7% (*v*/*v*) of glycerol. The samples were boiled for 10 min. and centrifuged for 7 min. at 14,000 rpm. The protein content in the supernatant was measured while using a modified Bradford assay [49] with bovine serum albumin (BSA) as the standard. The samples were electrophoretically separated (SDS-PAGE) on pre-made MINI-PROTEAN TGX Precast Gels (Bio-Rad, Hercules, CA, USA) gels, using a program of 18 mA, 30 W/1 gel, 30–110 V for 80 min. using a MINI-PROTEAN III (Bio-Rad). After electrophoresis, the proteins were transferred from the gel to a 0.45 µm PVDF (Bio-Rad) membrane using a MINI-PROTEAN III (Bio-Rad) apparatus and pH 8.7 transfer buffer [25 mM Tris, 192 mM glycine and 20% (*v*/*v*) methanol]. The membrane was subsequently incubated in TBST solution with 5% BSA and the primary antibody. The experiments utilized antibodies that were directed against the acetylated form of histone H3 (primary rabbit anti-acetyl-Histone H3 antibody, Merck, Darmstadt, Germany) (dilution 1:1000, 2 h), and then acetylated α-tubulin (primary mouse monoclonal anti-acetylated-tubulin antibody, Sigma-Aldrich, St. Louis, MO, USA) (dilution 1:1000, 2 h). The membrane was washed several times in TRIS buffered Saline with Tween 20 (TBST) buffer and then incubated in TBST and 1% BSA solution containing secondary antibodies (anti-rabbit and anti-mouse, respectively, Sigma-Aldrich, St. Louis, MO, USA) that were conjugated to alkaline phosphatase at a 1:1000 dilution for 1 h. Positive controls were also used, including histone H3 rabbit and tubulin alpha chain antibodies (Agrisera, Vännas, Sweden) dilution 1:10,000 and 1:1000, respectively, for 1 h. Immunodetection was performed while using the fast-BCIP/NBT solution (Sigma-Aldrich, St. Louis, MO, USA). Images of the stained gels were obtained and documented using an Image Scan and Densitometric Image Scanner III (GE Healthcare, Little Chalfont, UK). The level of acetylated protein in the images was quantified from three independent biological samples using Image Master 2D Platinum v. 7 (GE Healthcare, Little Chalfont, UK) software. The data are presented as relative units that are calculated as the intensity (density) and the area of the band. The proportions of acetylated histone H3 to non-acetylated histone H3 and acetylated tubulin to non-acetylated tubulin was calculated.

### 4.3. Analysis of Tubulin Cytoskeleton

The protocol that was described by Fischer and Timberlake [50] was used to visualize the, microtubule structure. Root fragments were fixed for 1.5 h at room temperature in a microtubule stabilizing buffer (PME) containing 50 mM piperazine-*N*,*N*′-bis[2-ethanesulfonic acid] (PIPES) (Sigma-Aldrich, St. Louis, MO, USA), 2 mM MgSO4 (POCH, Inc., Gliwice, Poland), 5 mM ethylene glycol-bis(2-aminoethylether)-*N*,*N*,*N*′,*N*′-tetraacetic acid (EGTA, Sigma-Aldrich) at pH 8.0 with 4% freshly prepared formaldehyde (*w*/*v*) (Polysciences, Warrington, PA, USA), and 2% dimethyl sulfoxide (DMSO, Sigma-Aldrich). The material was washed three times in PME (50 mM PIPES, 25 mM EGTA, 5 mM MgSO_4_) and then embedded in Steedman wax [51]. Ten µm thick cross-sections of root fragments were obtained and then placed on a glass slide coated with Mayer’s glycerol albumin. The wax was then removed from the sections using increasing concentrations of ethanol. The sections on the glass slides were subsequently rinsed in 0.01 M PBS. Afterwards, the sections were then treated with 0.01% acetylated bovine serum albumin (BSA-c) (Aurion, the Netherlands) in PBS for 10 min. and subsequently treated overnight in a humidity chamber with primary mouse monoclonal anti-α-tubulin antibody of high specifity to an epitope that was located at the *C*-terminal end of the α-tubulin (Sigma-Aldrich) diluted 1:500 in BSA-c/PBS, at 4 °C. The sections were then rinsed in PBS four times, followed by treatment with a secondary FITC-conjugated goat anti-mouse antibody (Sigma-Aldrich) and dilution of 1:200 in BSA-c/PBS for 2 h at room temperature. After rinsing five times in PBS, the material was mounted in Citifluor and observed using a Leica SP5 II confocal microscope. The sections were also stained with 4′,6-diamidino-2-phenylindole (DAPI) to visualize the nuclei. The frequencies of the cell division in the root cells has been also recorded (the number of analyzed cells in each root piece was 30).

### 4.4. Assessment of Cell Death (Nuclear DNA Fragmentation)

A TUNEL assay was conducted while using an in situ cell death detection kit (Fluorescein; Roche Applied Science, Penzberg, Germany) according to the instruction manual to assess DNA fragmentation. The root segments were fixed in 4% paraformaldehyde in phosphate saline buffer (PBS) and then dehydrated in increasing concentrations of ethanol in water (from 10 to 100%) and embedded in Technovit 7100. The root pieces were then incubated in 50 µL of the TUNEL reaction mixture. The solution was vacuum-filtered into the root fragments three times for 1 min. at 25 mmHg and then incubated for 60 min. at 37 °C in the dark in a humid atmosphere. The roots were subsequently washed and transferred to 1× PBS (pH 7.4) for destaining and observed using confocal microscopy (TCS SP5 II Leica, Wetzlar, Germany) while using an argon laser at 488 nm for excitation. TUNEL-positive nuclei were excited at 488 nm and detected at 505–540 nm. Grade 1 DNase I treated roots were used as a positive control. The quantification of TUNEL-positive nuclei was conducted to determine the timing of DNA cleavage after exposure to the various treatments that are described in the previous section.

### 4.5. Statistical Analysis

All of the experiments were repeated three times for each treatment. The effect of the different siderophores and the presence of iron was analyzed by either two- or one-way analysis of variance (ANOVA), followed by a Tukey’s HSD test. The differences between treatments were considered significant at *p* ≤ 0.05. All of the statistical analyses were conducted while using JMP v. 13 software (SAS Institute, Cary, NC, USA).

## 5. Conclusions

We investigated the interaction of *Pinus sylvestris* roots with mycorrhizal or parasitic fungi in phase 0, which is the stage preceding the direct contact of the fungus and root. A comparison of two experimental systems: (1) seedlings of pine seeded with fungus, which secretes metabolites to the substrate, and (2) pine seedlings treated with siderophores, enabled the determination of the significant effect of siderophores on root cell response. The reaction of roots to pathogenic fungal metabolites (cytoskeleton destruction, lack of cell division, root cell death) significantly differed from what was observed for the mycorrhizal fungus (slight changes in microtubule acetylation, cell division). At the same time, neither the parasitic nor the mycorrhizal fungi significantly modified the histone acetylation. Therefore, it does not appear that acetylation is the mechanism that is involved in the case of the initial stage of fungal/root interaction. It is likely that the root cell response is non-nuclear and it takes place at the cytoplasmic level (e.g., cytoskeleton modification). As previously concluded, bacterial hydroxamic siderophore–pyoverdine acting on iron imbalance switching between growth and immunity of plants [34] and it depends on the specific pathosystem and pyoverdine type, which is bound to iron disbalance [52]. In our studies, the form of a siderophore, bound or unrelated to iron, modulated the root response that was caused by the fungus. Therefore, the invasiveness of the fungus might be dependent on the availability of iron and the use of iron by binding to it with a siderophore. Siderophores (both pathogenic and mycorrhizal), which are secreted by fungi, seem to be crucial for root infection and they are important response for the establishment of a relationship consistent with the fungal life strategy. Thus, it appears that the manner in which the fungus interacts depends on the type and structure of the secreted siderophores, and can thus be predetermined in the phased before the partners come into direct contact.

Further research could reveal to what extent various types of hydroxamic siderophores that are associated with acetylation affect plant response. Additionally, further research could also help to determine whether acetylation is necessary for fungal entry into host tissues. Fast and reversible acetylation regulates the response to abiotic and biotic stress and might affect adaptation or lack of it to stress conditions. By explaining which of the enzymes that affect acetylation are activated in response to a specific pathogen, efforts to enhance plant’s resistance to pathogens, but still ensure the development of symbiosis with microorganism that support plant growth.

## Figures and Tables

**Table 1:** The results of an analysis of variance (ANOVA) on the effect of siderophore variables on the ratio of acetylated α-tubulin to non-acetylated α-tubulin (acTub/Tub), acetylated histone H3 to non-acetylated histone H3 (acH3/H3), percentage of cell division, and percentage of cell death. Siderophore variables were structurally-different siderophores (SIDER), the siderophores ability to bind iron (DES), and their interaction.

Independent Variable	df	acH3/H3		acTub/Tub		Percentage of Cell Division		Cell Death	
		F	*p*	F	*p*	F	*p*	F	*p*
Siderophores ability to bind iron (DES)	1	0.36	0.559	30.22	*0.001*	28.99	*0.001*	0.001	0.975
Siderophores compounds (SIDER)	2	5.55	*0.020*	8.15	*0.006*	95.59	*0.001*	22.77	*0.001*
SIDER×DES	2	0.21	0.817	14.44	*0.001*	98.11	*0.001*	6.42	*0.003*

Values presented in italics are statistically significant while non-italic values indicate a lack of statistical significance. The probability of mistakenly rejecting a null hypothesis that is actually true was estimated at *p* = 0.05; df—degrees of freedom, F—variation between sample means to variation within the samples.

## Supplementary Materials

Supplementary materials can be found at https://www.mdpi.com/1422-0067/20/23/6099/s1.
