# Supplementary figures and images for: The Effect of Hydroxamic Siderophores Structure on Acetylation of Histone H3 and Alpha Tubulin in Pinus sylvestris Root Cells
Source: Int J Mol Sci. 2019 Dec 3;20(23):6099. doi: 10.3390/ijms20236099 (PMC6928989; doi:10.3390/ijms20236099)

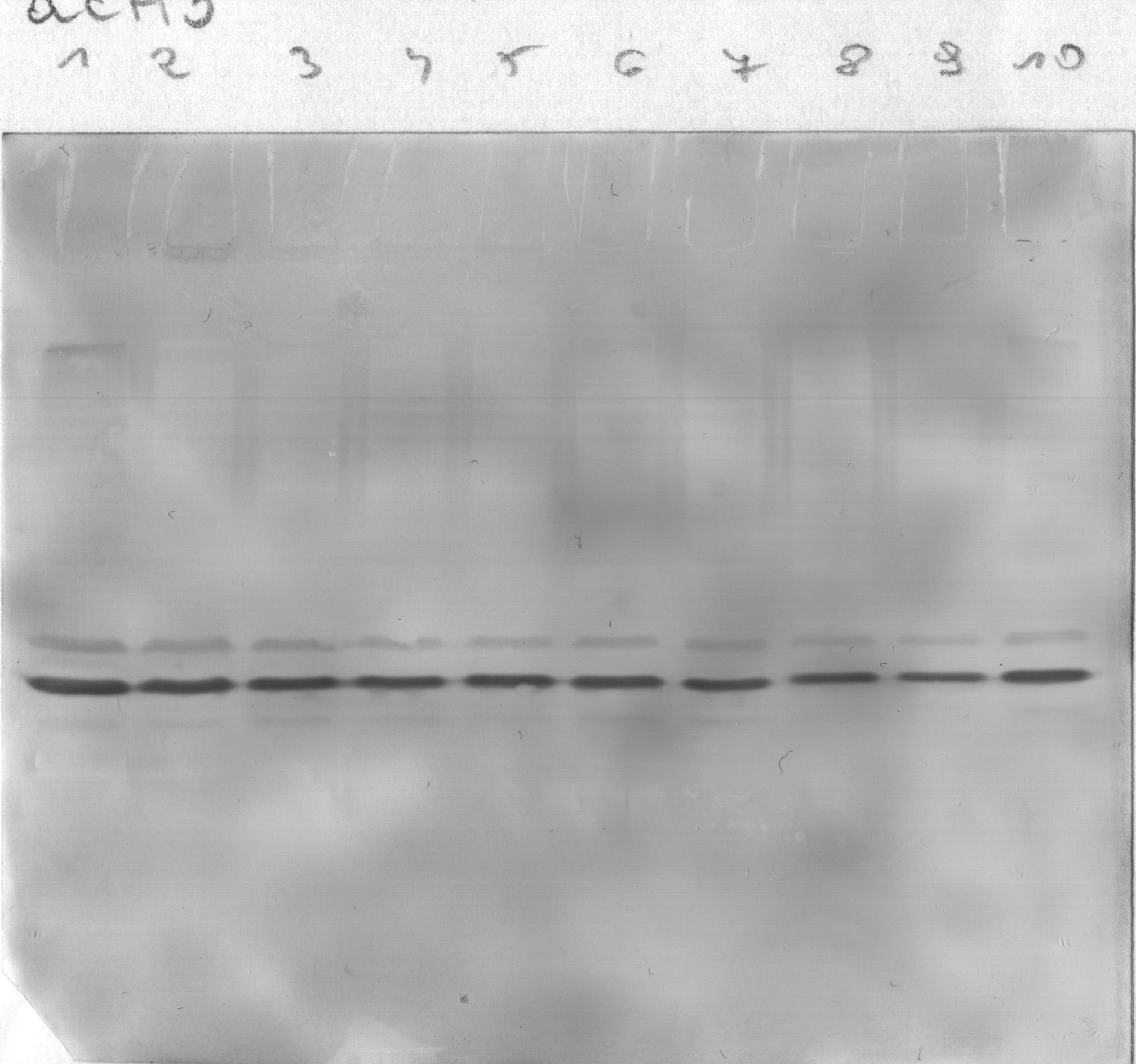

Supplement: Supplementary file 1 [file ijms-20-06099-s001.zip › ijms-623756-supplementary/to Fig. 1_acH3.tif]

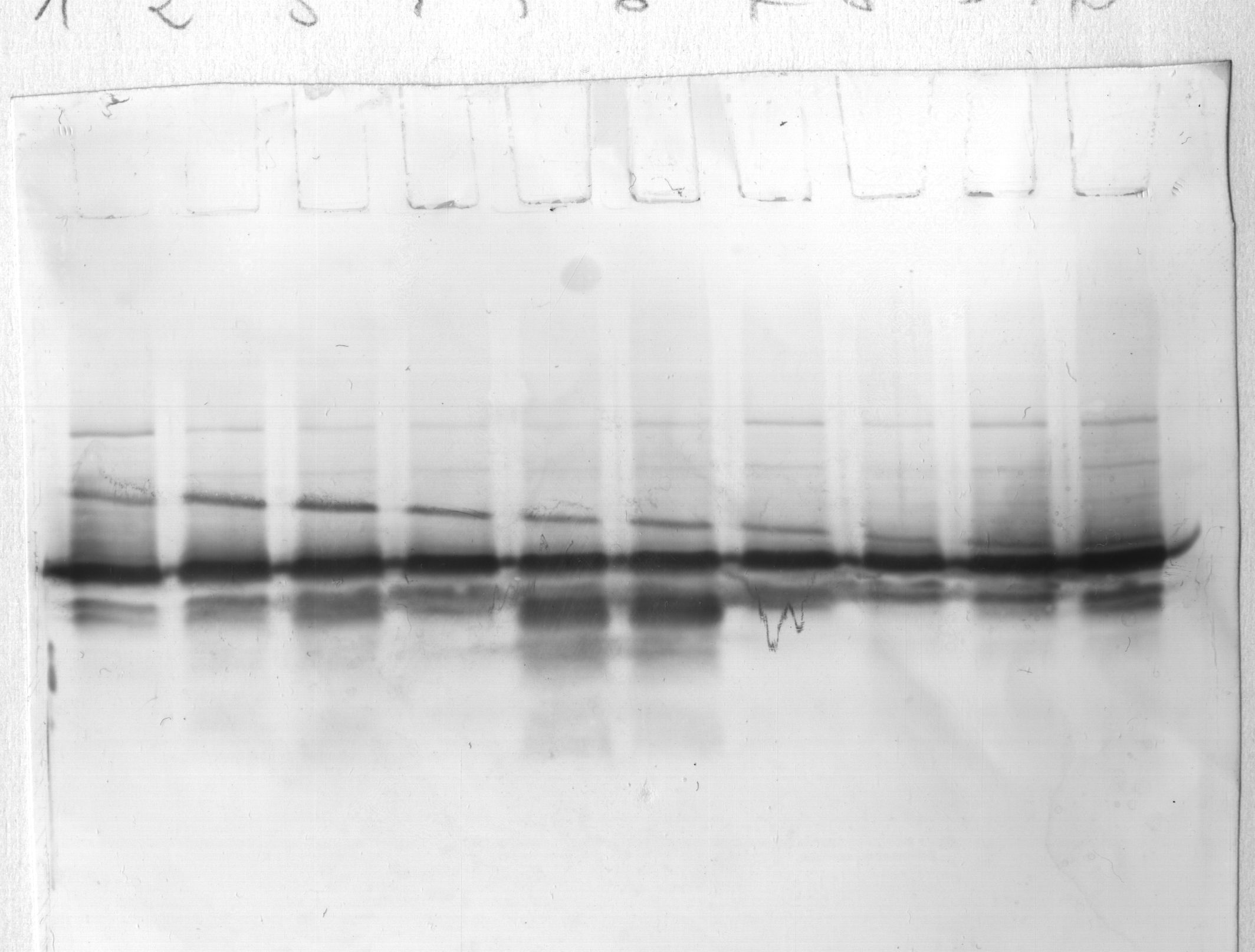

Supplement: Supplementary file 1 [file ijms-20-06099-s001.zip › ijms-623756-supplementary/to Fig. 1_H3.tif]

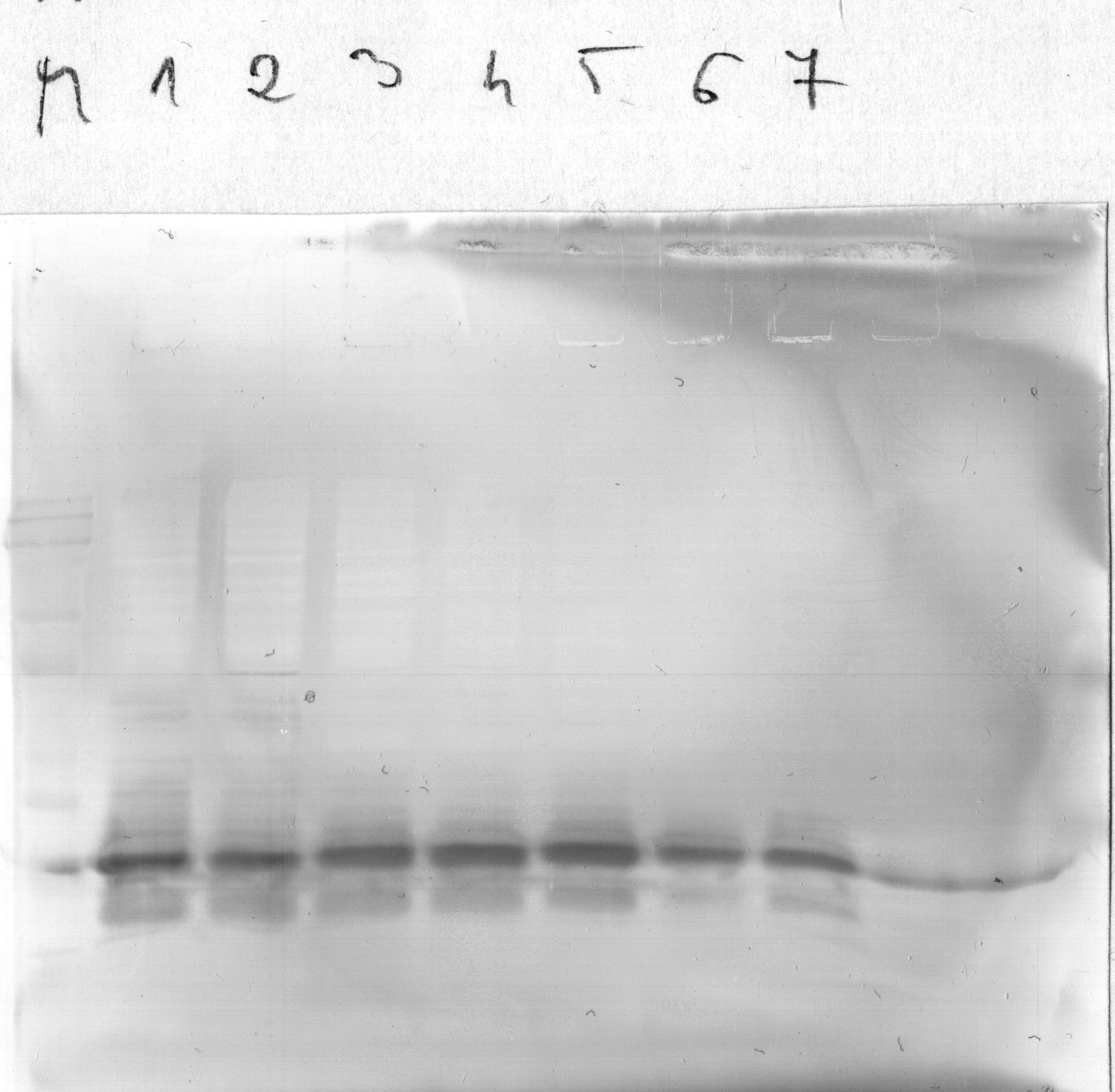

Supplement: Supplementary file 1 [file ijms-20-06099-s001.zip › ijms-623756-supplementary/to Fig. 2_H3.jpg]

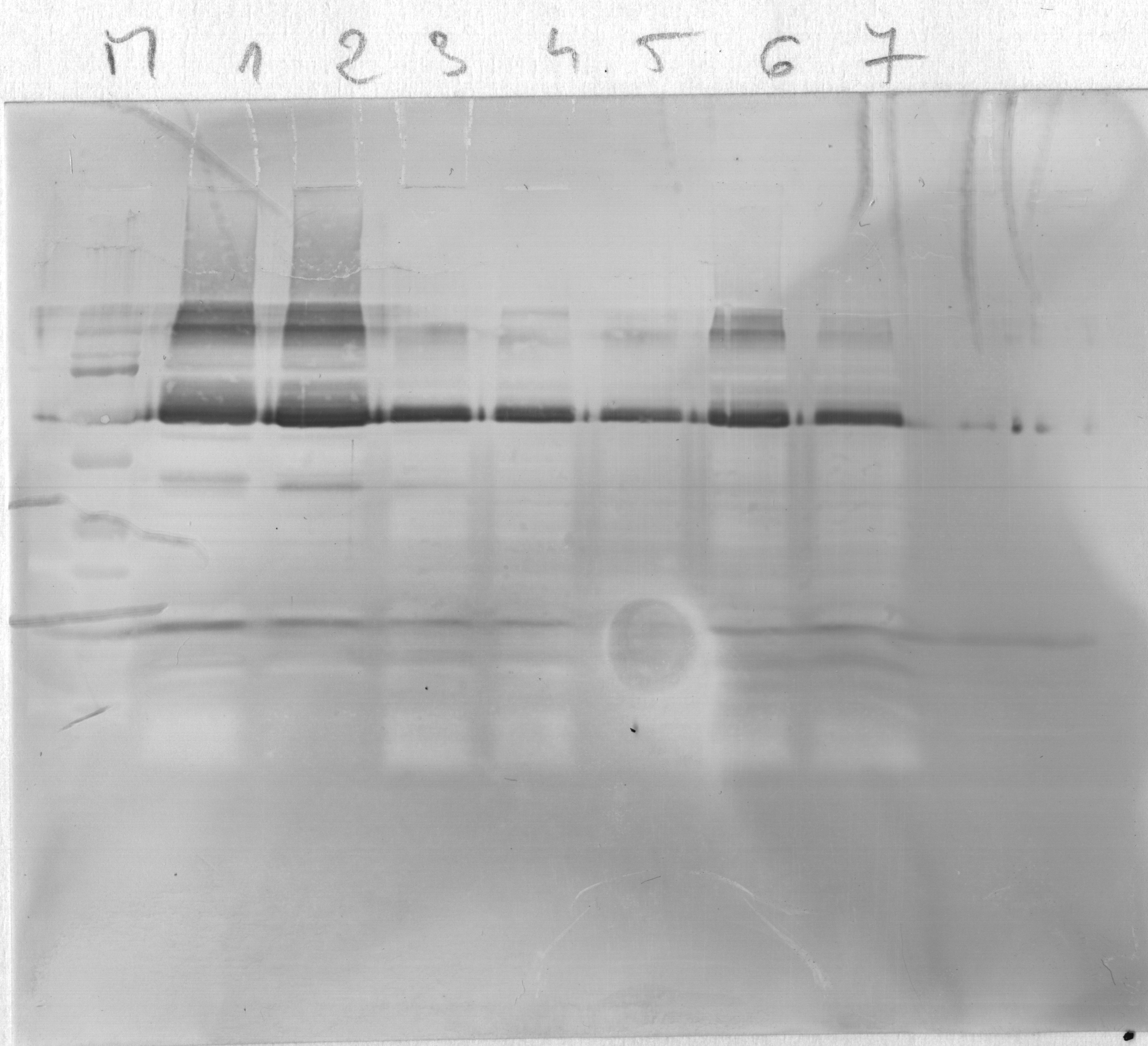

Supplement: Supplementary file 1 [file ijms-20-06099-s001.zip › ijms-623756-supplementary/to Fig. 4_Tub.tif]
